# Evaluation of additive manufacturing of sand cores in terms of the resulting surface roughness

**DOI:** 10.1016/j.heliyon.2022.e10751

**Published:** 2022-09-25

**Authors:** Martina Gawronová, Petr Lichý, Ivana Kroupová, Tomáš Obzina, Jaroslav Beňo, Isabel Nguyenová, Václav Merta, Jan Jezierski, Filip Radkovský

**Affiliations:** a Department of Metallurgical Technologies, VSB-Technical University of Ostrava, Faculty of Materials Science and Technology, 17. Listopadu 2172/15, 708 00 Ostrava, Czech Republic; b Brembo Czech, S. R. o., Na Rovince 875, 720 00 Ostrava, Czech Republic; c Department of Foundry Engineering, Silesian University of Technology, Faculty of Mechanical Engineering, Ul. Towarova 7, 44-100 Gliwice, Poland

**Keywords:** Moulding mixtures, Surface properties, 3-D printing, Casting

## Abstract

Obtaining a good surface finish on casting is challenging and depends on the dimension of the sand particles and the processing method of the mold. Evolving modern trends in mould and core production as a binder jetting technology is an option and it is more than desirable to evaluate and remove any possible negative effects. The aim of this study is to compare the influence of furan no-bake technology and 3D printing method on the surface quality of cores and cavities formed in aluminium alloy castings. In addition to the sieve analysis and mechanical properties of the moulding mixtures, the roughness (Ra, Rz) of the cores and resulting casting surfaces of individual samples were compared in this study.

## 1 Introduction

The surface of the product determines the final appearance, therefore roughness can be considered as a certain reflection of surface quality. One of the factors influencing the appearance and quality of the surface is undoubtedly the influence of the coremixture, both in terms of their mechanical properties and the quality of the processing of the mixture itself. Binder systems for the production of cores achieve different characteristics both in primary production (moulding or shooting the mixture) and in metal casting [1]. This may affect the surface properties of the casting from the core side. The inner surface of the casting exposed, for example, to flowing liquids (water pumps, nozzles, etc.) is significantly involved in the behaviour of the liquid flowing in the casting cavities, and thus hydraulic losses can occur. Therefore, the aimis to achieve the finest possible surface. Cores of complex shape can be produced using an inorganic binder system. These cores are characterised by sufficient strength and abrasion resistance and the resulting high quality and smooth casting surface. On the other hand, the problems with high temperature expansion of the cores and poor collapsibility and so deteriorated decoring need to be considered [2, 3, 4]. Organic binders are often used as a solution for such production. In the study the author [5] recommends to use cores bound with resin of the RCS (resin coated sand) type, which guarantee a very good surface with a minimum of defects in castings of grey cast iron and nodular cast iron. However, the problem with this system is impaired collapsibility in case of alloys with lower melting temperatures (aluminium or magnesium alloys), and thus low core heating to achieve improved collapsibility. In the automotive and other industries, Al–Si alloys, so-called silumines, are used as universal materials for the production of complex castings (such as pump bodies, transmission cases, housings) because of their good technological and foundry properties as low density combined with sufficient strength [6, 7, 8]. One of the most commonly used alloys is hypoeutectic alloy A356 because of its good castability [9], excellent mechanical properties [10, 11], corrosion resistance [12, 13] and good dimensional stability and strength at different temperatures [14]. With aim to improve collapsibility, other authors report the suitability of using binder systems based on furan [15, 16], polyurethane resin [17, 18] or alkaline phenolic resin hardened by CO_2_ [19] in the aluminium foundry. Although the mechanical properties are lower than that ones of RCS bound mixtures, moulds and cores bound with furan resin can be hardened at room temperature with achieving sufficient strengths. Also, their very good collapsibility after thermal exposure defines their advantages for shape-complex components with precise dimensions, especially in the automotive industry [20, 21]. Furthermore, technological, ecological and economic aspects of production such as good sand reclamation, lower investment requirements for technology (conventional mixers, non-metal core boxes), higher economics of core production (lower acquisition costs per mixture, lower energy consumption due to the elimination of the need for preheating the core boxes) [20, 22, 23], reduction of carbon emissions achieved by reducing liquid metal consumption through efficient mould design and minimal post-processing after casting [24, 25], can represent a certain operational advantage and with a suitably chosen sand granulometry a quality surface can be achieved [21].

In addition to the conventional production methods as no-bake technology, there is now an effort to produce cores and moulds using new methods. One of them is the increasingly widespread and popular the 3D sand print (3DSP) of moulds and cores using binder jetting technology. The technological principles of the production of cores and moulds using 3D sand print have already been studied by many authors [22, 26, 27, 28, 29], when they agree that the use of such systems is possible in practice. 3DSP technology enables a variety of production with the possibility to produce high-precision and shape-complex parts with accuracy of ±0.5 mm or an error of less than 0.3%, and thus find application in prototype production, automotive and in the design of innovative production processes in the field of metal foams [30], or experimental production of rib enforced shell moulds for controlled and accelerated cooling of aluminium alloy castings [31, 32]. In other words, the production of core boxes, and the associated dimensioning, and the production of metal mould [22, 26] fall away.

Different types of 3D sand printers use different chemical sand curing systems. No-Bake systems based on furan and phenolic resin, or inorganic alkaline silicates are most commonly used for the production of 3D sand printed cores. It is also possible to use a system of phenolic resin bonded with heat. Very fine fractions of silica sand or synthetic ceramic sand are often used as a sand [26, 33]. Unlike other types of 3D sand printers using one-component binder system that require more binder, ExOne printers use a two-component binder system that is very similar to conventional furan no-bake technology [34].

**Figure 1:**
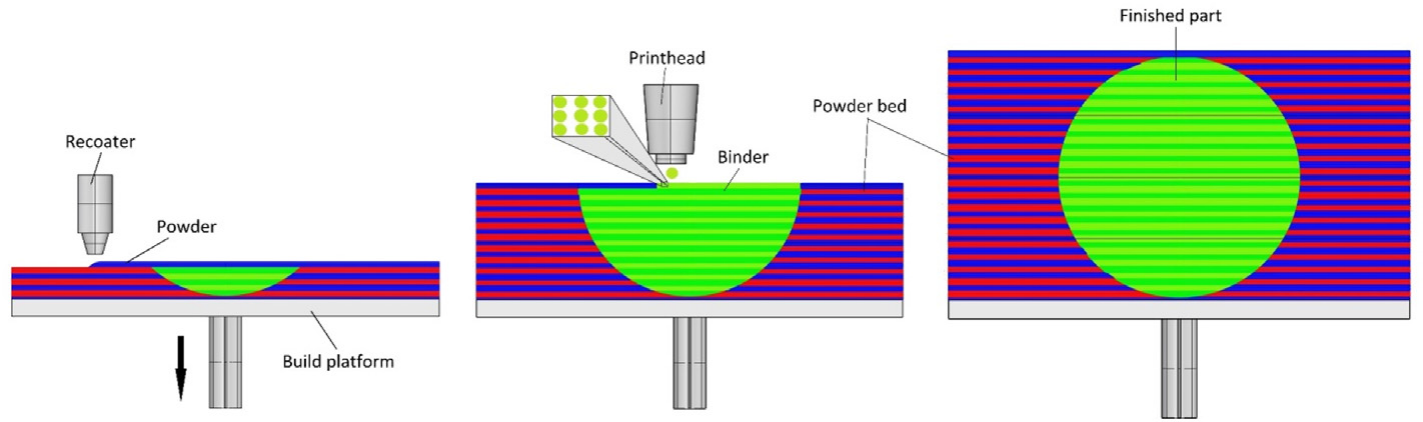
Binder jetting technology schema.

3D printing of sand moulds is, in principle very similar to the well-known 2D printing on paper using an inkjet desktop printer. Figure 1 schematically shows the binder jetting technology process. A job box is placed on the printer platform in which a recoater evenly spreads 0.28–0.5 mm thin layer of powder particles, such as silica sand or ceramic particles, mixed with a catalyst. Droplets of liquid binder are then applied to this layer using a print head moving at the speed of 60–80 mm s^−1^. The binder is only dispensed at certain positions and on contact with the catalyst, the chemical curing process starts at that point. The remaining places in the powder bed remain filled with loose sand only. The platform is them moved downwards a predefined distance, the recoater dries the bed and applies a new layer of powder with catalyst over the already cured bed. As the recoater moves, the new layer of sand is also gently compressed. The build speed achieved is usually 60–85 l h^−1^. This process is repeated layer by layer until the object is completed to the designed 3D specification. The finished part is the removed from the powder bed [23, 24, 35, 36].

**Figure 2:**
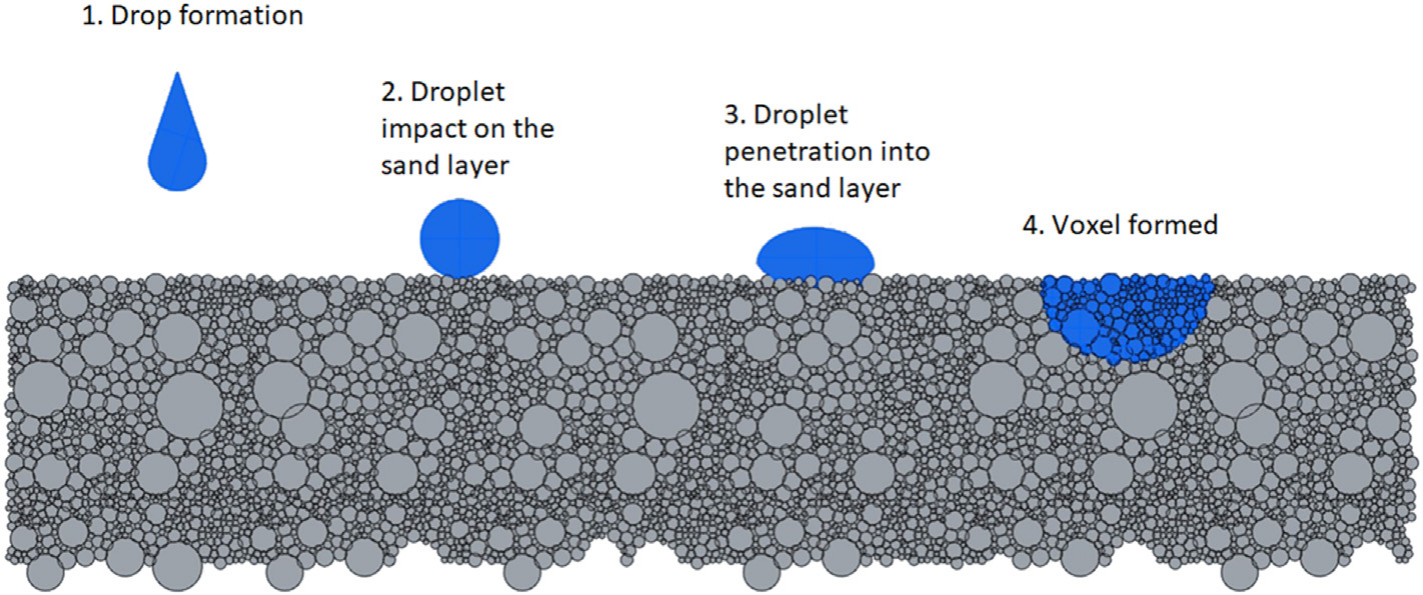
Dropping of binder into a powder.

Several different authors [28, 37] observed poorer surface quality of castings after using 3DSP moulds or cores, either due to higher surface roughness or the presence of minor surface defects. The main reason appears to be the excess binder with which 3D sand printers generally work. As can be seen from Figure 2, the powder contains particles of different sizes and shapes (granulometric composition of the basic sand), which the binder must be able to coat at the desired layer depth. Therefore, the application of the liquid binder must be precise, the individual droplets must be very fine, and they must only flow in a vertical direction to the surface of the powder, so that the binder does not run over the powder. There is also a need for sufficient hardening of the binder before applying an additional layer of sand (recoating process) to prevent the spread of new sand on still moist particles [36, 38, 39]. Also, the newly applied layer must be compressed with a sufficiently large force to reduce the amount of pores in the mixture [38]. The porosity of the mixtures is important in predicting the permeability of the mixture, while the pore size affects the penetration of the metal into the foundry mould and also directly affects the resulting roughness of the casting or the occurrence of foundry defects. Thus, the finer and more angular the grains, the higher the porosity of the mixture [40, 41].

The binder dosage is generally above the dosage level for no-bake core preparation methods (binder content <1%). There have been several studies [35, 37, 42] investigating the effect of binder dosage and saturation on mechanical properties in terms of strength, permeability and dimensional compliance. This author states [43] that the 3D sand print technology used by them works at a binder content of 1.6–1.8% (for furan), another source reports a furan dosage of 1–3% [39] and another author [37] lists contents approaching up to 8% of a binder. Generally the mechanical strength increases with increasing amount of binder. However, the higher binder content leads to a deterioration of the mould collapsibility after pouring, as the strength of the mould remains even after heat exposure [44]. It has been also found that a higher binder dosing rate in 3DSP may cause impaired mould or core shrinkage and permeability, higher gas development, resulting in deterioration of surface quality [28, 38] or formation of gas defects [35]. On the other hand, the deteriorated roughness of the surface can also be caused by insufficient compression of the newly applied layer of the sand mixture and catalyst, which is usually caused by the high speed of spreading the new layer by the recoater [26, 29, 38]. Since in the 3DSP technology there is also a requirement for high production speed of the piece, this can lead to uneven compaction of the sand in the bed and consequently to the reduction of the density of applied sand due to higher porosity [37].

Obtaining a quality cast surface without machining is challenging in the case of water pump casting or in automotive industry. The parts are mainly manufactured from A356 alloy in combination with complex cores with organic binders, and additive manufacturing technology of cores is also used. This study focuses on the resulting inner surface roughness of A356 aluminium alloy casting where different cores were used for production. Furthemore, the mechanical properties of cores made by furan no-bake and 3D sand print technology will be investigated, as well as the influence of production parameters on the surface quality of the castings.

## 2 Materials and methods

**Figure 3:**
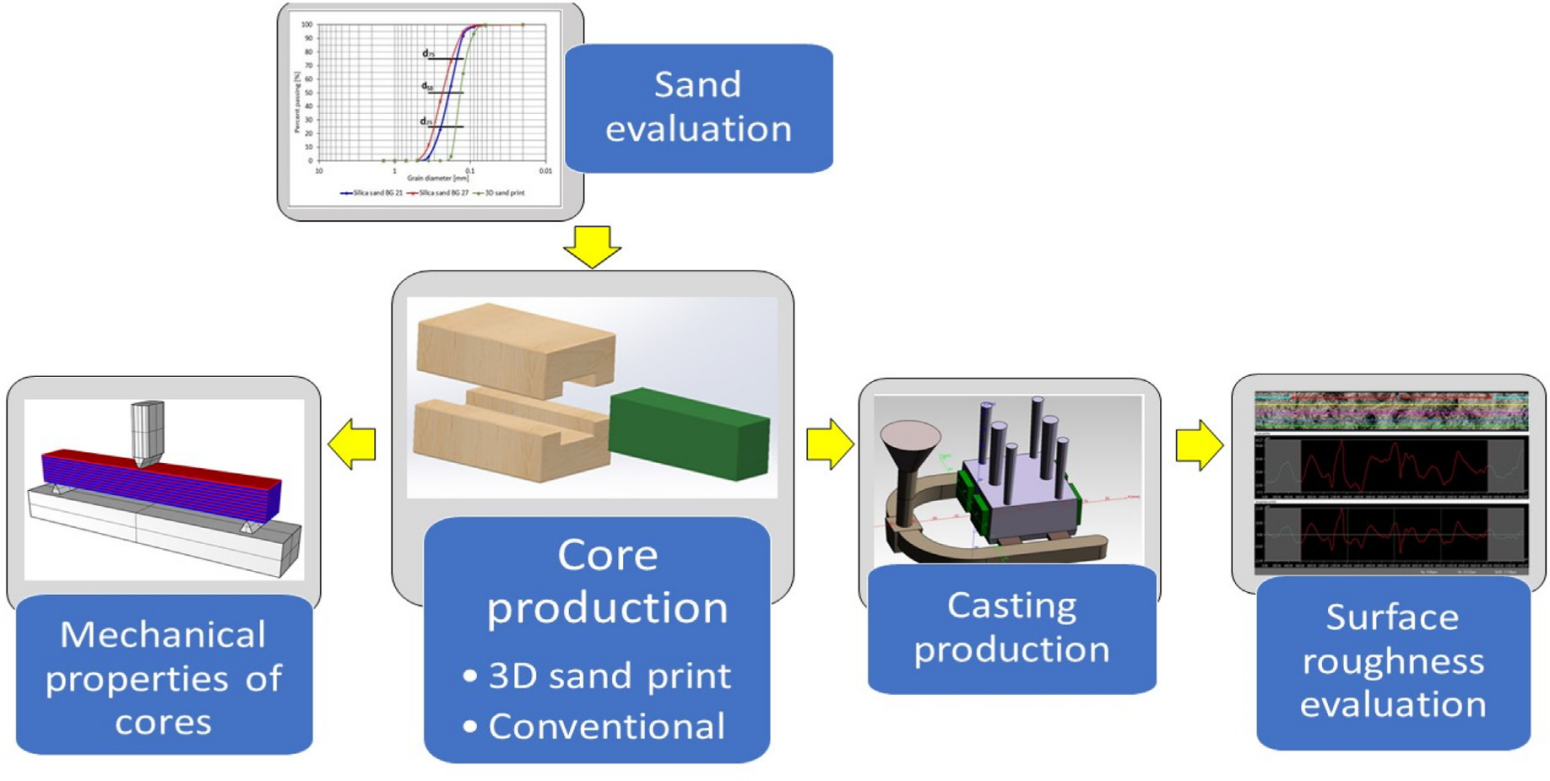
A schematic of used methodology.

A schematic of the test sample production methodology and the order of the individual measurement groups can be seen in Figure 3.

### 2.1 Sand characterisation

Silica sand from a locality Biala Góra in Poland, with two different mean grain sizes, marked by the supplier as BG 21 and BG 27, was used for the production of furan no-bake cores. Guaranteed mean grain size of sand with the designation BG 21 was d_50_ 0.21 mm ± 0.02 mm, the second BG 27 was d_50_ 0.27mm ± 0.02 mm mainly used for the technology with organic resins. Mean grain size 0.21 mm was chosen despite the fact that it was the sand rather used for green sand mixtures, as it represented the size intermediate step between the granulometry for furan technology and for 3Dsand print technology.

A detailed overview of the granulometric composition based on clay wash analysis according to ASTM C117-13 and sieve analysis according to ASTM E11 were performed. The mass of each sample was 50 g. Laboratory sand siever (LPzE-2e, Multiserw-Morek, Poland) equipped with compliant sieves was used. Shaking time was set on 10 min, vibration 50 kHz, amplitude was adjustable from 0 to 2.5 mm. Grain fineness number according to American Foundry Society (AFS) was determined from a percentage of sand fraction captured on each sieve using a multiplier. Cumulative curves of granularity were evaluated and criteria of the granulometric composition as values for d_25_, d_50_ and d_75_ and the Homogeneity degree S were calculated (equation 1).

(1) $$S=\left(d_{75}/d_{25}\right)\cdot 100\;\left(\%\right)$$

where d_25_ and d_75_ for mesh diameters corresponding to 25 and 75 % of the total sand mass (after washing off the particles less than 0.02 mm).

The Criterion of the grain-size distribution probability log W (equation 2) was also determined from the sieve analysis. Compared to the evaluation criterion of the grain fineness number according to AFS, this analysis provides an overview of sorting level (monofractional or polyfractional sand).

(2a) $$N_{i}=\left(m_{i}/m_{p}\right)\cdot 100\;\left(\%\right)$$

(2b) $$logW=100\cdot log\left(100\right)-\sum N_{i}log\left(N_{i}\right)$$

where N_i_ for fraction captured on the sieve (%), m_i_ for the mass of sample sand on sieve (g) and m_p_ for the mass of sample sand after washing off clay particles below 0.02 mm (g).

Initial data of particle size distribution of sand used for 3Dsand print resulted from sieve analysis was provided by the manufacturer and was further used to determine the log W, S and cumulative curve of granularity.

### 2.2 Core production technology parameters

**Figure 4:**
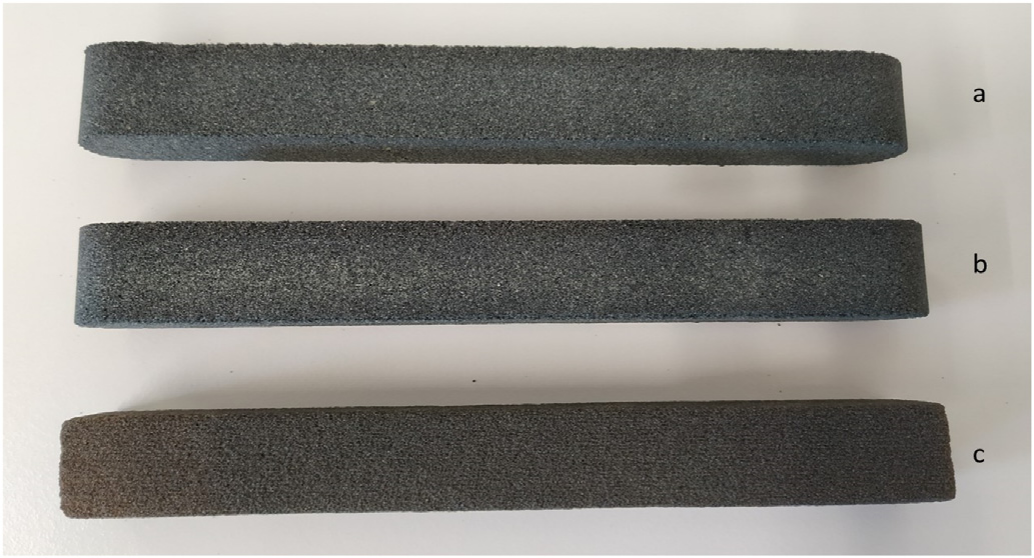
Furan no-bake test samples: (a) silica sand BG 21, (b) silica sand BG 27, (c) silica sand used for 3D sand print.

Furan no-bake core production simulated usual production dosing of 1 % of binder per sand mass and 35 % of the catalyst per resin mass (0.35 % of the catalyst per sand mass). The mixture was mixed on a laboratory paddle mixer. Firstly, the sand-catalyst mixture was mixed for 1 min for curing homogeneity, then after adding resin binder to the mixture, the mixture was mixed for another 1 min. Then the mixture was compacted using a laboratory sand rammer (LUA-2e/Z, Multiserw Morek, Poland) in order to maintain constant conditions of core production. The mixed mixture was poured into 22.5 mm × 22.5 mm x 170 mm steel multi-cavity core box and subsequently compacted by three times hitting of the standard sand rammer with constant impact energy of 3.3 J Figure 4 shows samples made by furan no-bake technology and 3D sand print.

3DSP core samples were produced by local commercial supplier using the binder jetting technology on the ExOne's 3D sand printer, which was used in studies by other authors [22, 27, 30]. The size of printed sample cores was 22.5 mm × 22.5 mm x 170 mm (Figure 4). Silica sand with d_50_ 0.14 mm (hereafter referred to as 3DSP sand) was used. Furan resin supplied by ExOne was used, which was hardened using a catalyst based on paratoluenesulphonic acid. According to the parameters recommended by the manufacturer of the 3D sand printer the catalyst dosage was 0.21 % per sand mass. The dosage of furan resin was not precisely defined by the supplier, as well as the exact speed of printing head and recoater. The input amount of furan resin in the used test cores was therefore reversely determined in the laboratory by loss of ignition test and it was around 1.25% ± 0.02 % per sand mass. Basic parameters of binders and catalysts used are given in the table below (Tables 1a and 1b)

**Table 1a:** Basic parameters of binders.

Component	No-bake	3D sand print
Binder addition [wt. %]	1.0	1.25
Furfurylalcohol [%]	Min. 75	Min. 70
Phenol [%]	<1	<1
pH [-]	6–8	6–8

**Table 1b:** Basic parameters of catalysts.

Component	No-bake	3D sand print
Dosing to the sand content [wt. %]	0.35	0.21
Paratoluenesuphonic acid [%]	<65	30–70
Sulphuric acid [%]	<2	Max. 5
pH [-]	<1	<1

### 2.3 Experimental procedure and measuring

• Transverse strength

**Figure 5:**
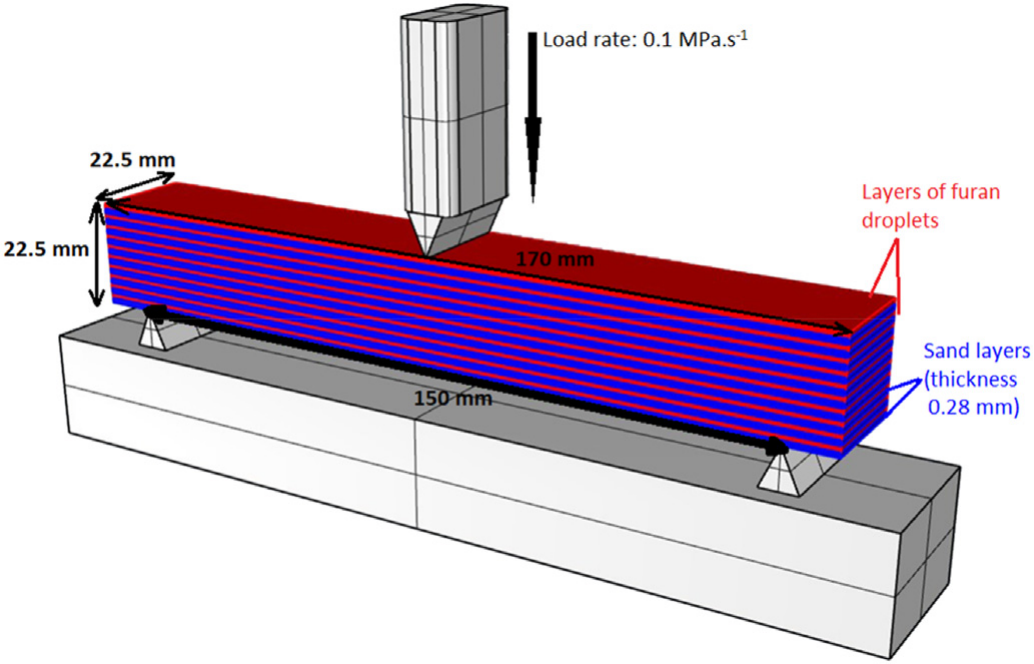
Schematic of 3D sand printed test specimen during 3-point bending strength measurement (Inspired by [38]).

The mechanical strength of the cores was characterised by a measurement known as 3-point bending strength and was carried out on the device for testing the strength of moulding sand (LRu-2e, Multiserw Morek, Poland) at room temperature. Constant load rate was 0.1 MPa s^−1^. Three measurements were made for every core type. The size of each specimen was 22.5 mm × 22.5 mm x 170 mm a were taken to measurement 24 h after curing (Figure 5).

• Abrasion resistance

Abrasion resistance was evaluated on samples taken after mechanical strength measuring and was carried out on a laboratory device with a rotating wire basket. The sample was placed in a perforated basket with a diameter of 180 mm, and it was subsequently abraded for 60 s at 57 rotations per minute. The resulting decrease in mass with a comparativeness to the original mass of the measured sample gives abrasion resistance percentages.

• Loss on ignition

The crushed cores were dried out into laboratory drier at 105 °C to remove all moisture. Then approximately 3 g samples in annealed ceramic crucibles were placed in muffle furnace heated at 900 °C for 2 h. Loss on ignition directly proportional to mass loss caused by volatilization of organics, in this case organic binders, and was determined by means of the difference in mass of the pre-dried samples before and after the ignition. Measuring was performed on the laboratory scale with sensitivity of 0.0001 g.

• Relative porosity of sand core

The relative porosity of cores was determined from the obtained sample densities and Eq. (3).

(3) $$m=\left(\frac{\rho_{1}-\rho_{2}}{\rho_{1}}\right)\cdot 100\;\left(\%\right)$$

where ρ_1_ for the specific mass of sand (kg^.^m^−3^) and ρ_2_ for density of compacted mixtures (kg^.^m^−3^).

### 2.4 Preparation of test castings

**Figure 6:**
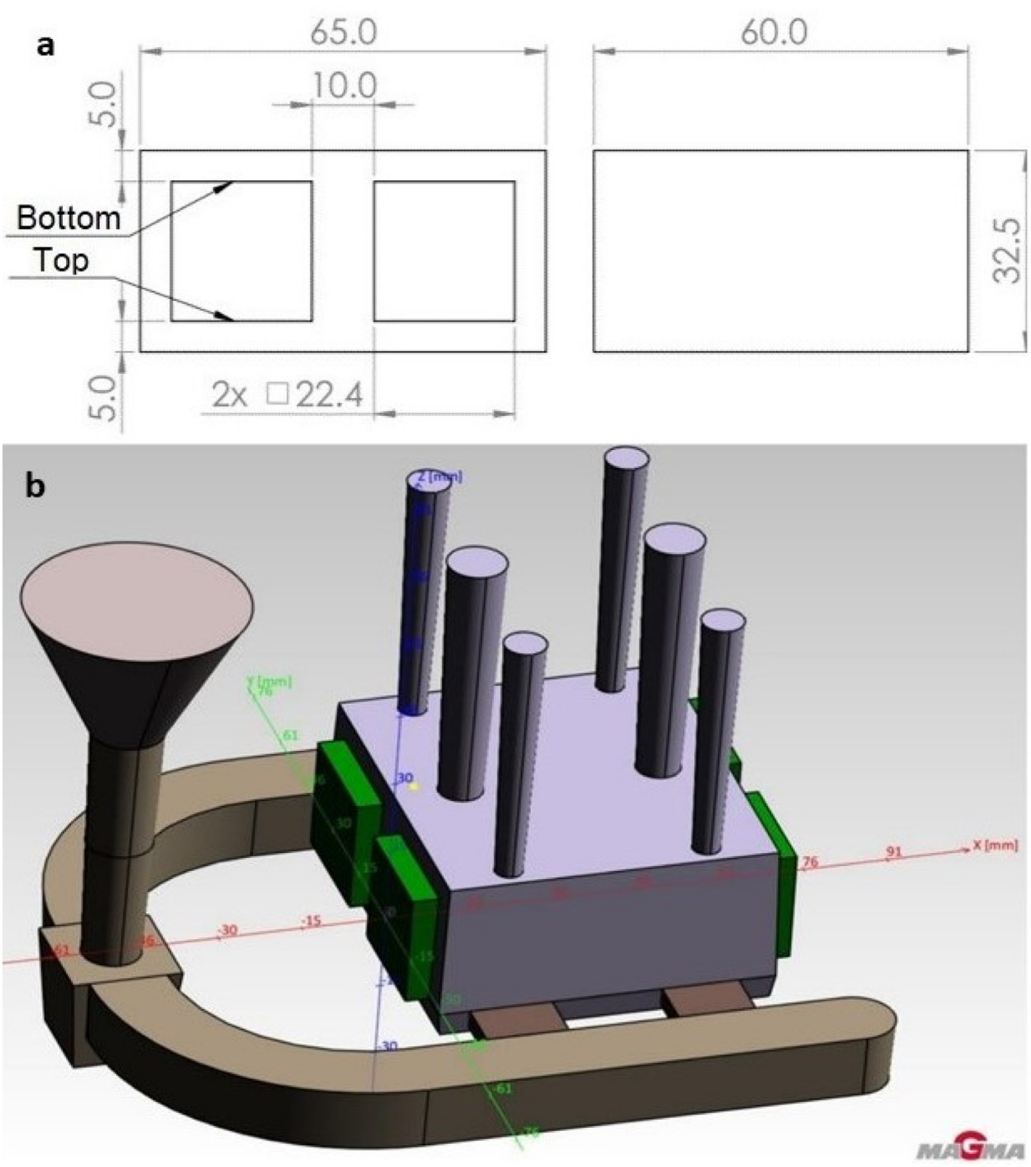
(a) Casting drawing. (b) 3D model of casting with cores obtained from MAGMA simulation program.

The production of test castings took place under laboratory conditions, see Figure 6 3D model of casting. The area of the casting marked as “Top” was created by the lower part of the core and represents the upper inner surface of the casting. The area marked as “Bottom” was created by the upper part of the core and represents the bottom inner surface of the casting. The mould was made using a no-bake geopolymer-based binder technology with the trade name GEOPOL. Tested cores with an original length of 170 mm have been shortened to 70 mm according to the casting design. The A356 aluminium alloy in a pure state was chosen for research purposes, without prior melting, with a guaranteed chemical composition. The casting temperature was 720 °C with a casting time of 4 s.

### 2.5 Determination of surface roughness of cores and castings

**Figure 7:**
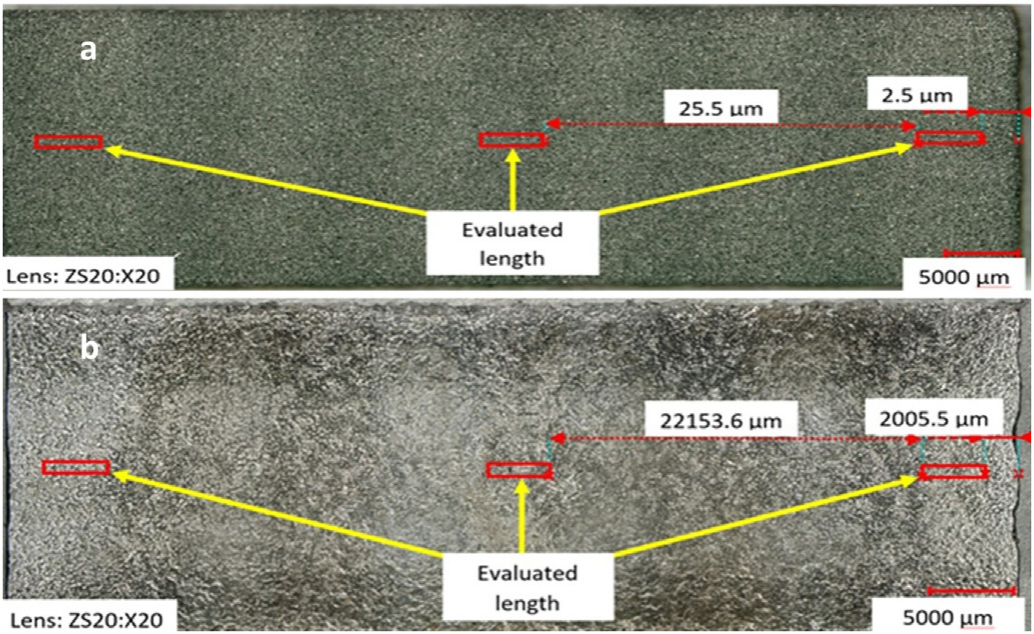
Determination of measured areas: (a) on the sand core, (b) on the casting surface.

The evaluation of roughness was carried out on digital microscope (OM, VHX 6000, Keyence, Japan) with a zoom range of 20x up to 2000x. Evaluation of roughness was carried out on photographs that were made up of partial figures using the "stitching" function to match the length for measuring of roughness and evaluated according to the ISO 4288 standards. Evaluated length values have always been taken from three “points”, red rectangles, see Figure 7 for sand core and casting surfaces.

The difference in measuring the roughness of sand cores, and by far grainy materials, compared to solids (castings) was the need to set the maximum (up) and minimum depth (down) of the sharpness, which was set to 500 μm.

The core surface roughness and internal surfaces (top and bottom) of the casting created by copying the core surface were measured. The castings were left in rough state without any machining,Figure 6 shows these areas with the terms "Upper" and "Bottom" which will be evaluated separately because the lower surfaces of the casting due to gravity achieve better surface quality than the upper surfaces.

## 3 Results

### 3.1 Basic parameters of the used sands

**Figure 8:**
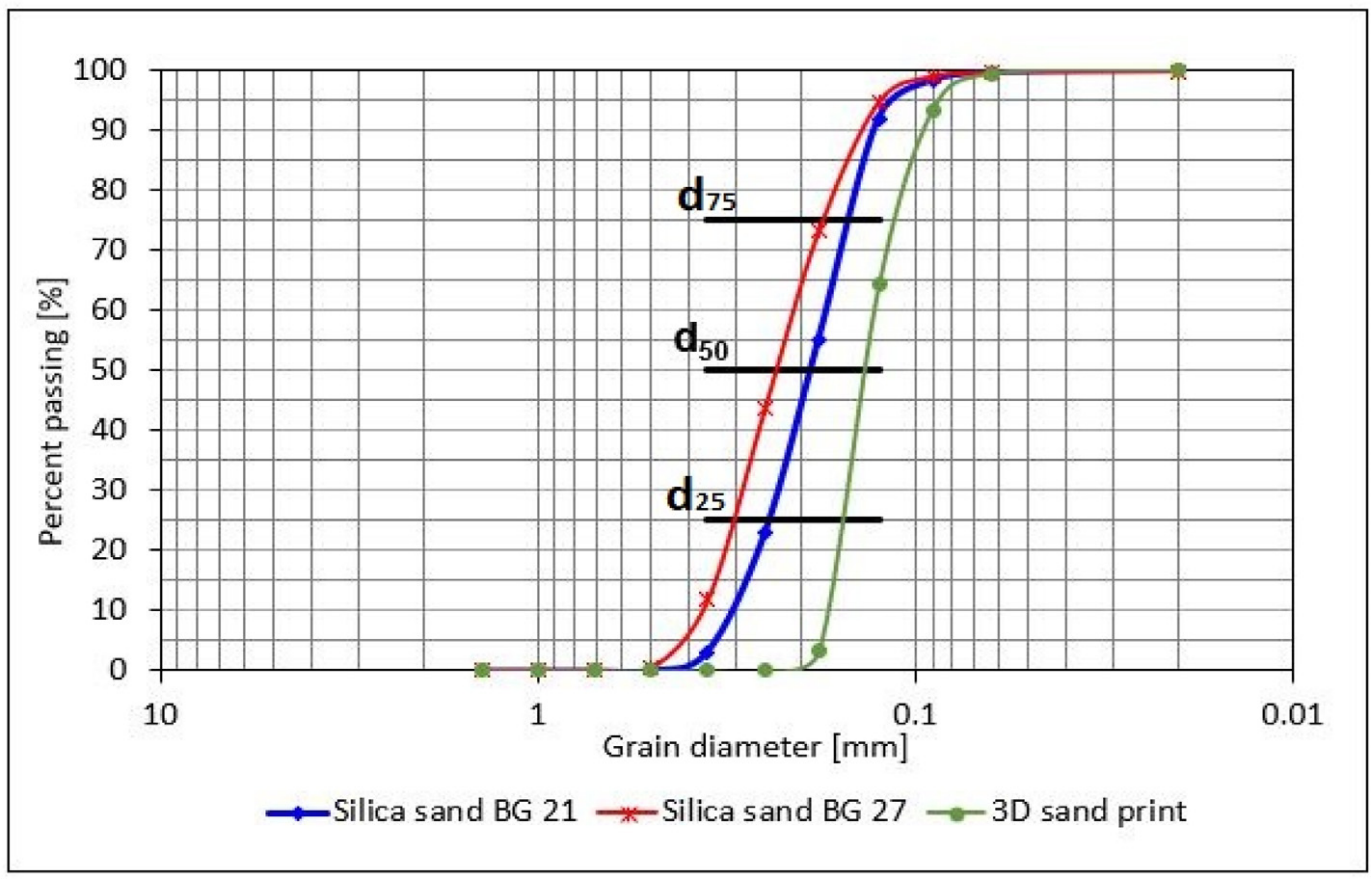
Comparison of cumulative curves of granularity for all tested sands.

The granulometric composition of all the used sands was determined using the sieve analysis. From the obtained values the AFS number together with Criterion of the grain-size distribution probability log W was determined, and cumulative curves of granularity for individual sands were compiled (Figure 8). The values for d_25_, d_50_ and d_75_ were subsequently read from them and the homogeneity degree S was determined. The particle size distribution is included in Table 2 together with the other criteria.

**Table 2:** Particle size distribution and basic parameters of test sand specimens.

Mesh size [mm]	Silica sand BG 21 [retained %]	Silica sand BG 27 [retained %]	3D sand print [retained %]
1.4	0	0	0
1	0	0	0
0.71	0	0	0
0.5	0.06	0.48	0
0.355	3.12	11.29	0
0.25	19.65	32.01	0
0.18	32.35	29.56	3.50
0.125	36.64	21.27	60.70
0.09	6.37	4.17	29.10
0.063	1.31	0.73	6.00
Pan	0.34	0.17	0.70

AFS Number	69	60	97
d_25_ [mm]	0.24	0.30	0.16
Mean grain size d_50_ [mm]	0.19	0.23	0.14
d_75_ [mm]	0.15	0.18	0.11
Log W [-]	61.33	63.97	41.49
Homogeneity degree S [%]	62.50	60.00	68.75

The measured results indicate that the sand used for 3DSP showed the log W value by 35 % lower than the BG 27 sand and by 32 % lower than the BG 21 sand, and thus the log W value for the 3DSP sand was closer to 0. It can therefore be concluded that the sand supplied for the needs of 3DSP sand was very sharply sorted compared to the sands used for furan no-bake core production, mainly containing grains of the same size, and approached rather monofraction with its properties. This conclusion is consistent with the set cumulative curves of granularity, where it is evident that the sand cumulative curve for 3DSP has a steeper increase. On the contrary, the curves for BG 21 and BG 27 sands have mainly fractions with different grain sizes, especially in case of BG 27 sand, which has the highest log W value and is more polyfractional.

The values of mean grain size d_50_ read from the cumulative grain curves for each sand correspond to the supplied sand parameters.

Since natural silica sands were used, it was also possible to evaluate the homogeneity degree S, for which it is valid that the sand is the more uniform the closer the S is to value 1 or 100 % respectively, and vice versa. The most homogeneous sand can again be considered the 3DSP sand due to the high degree of sorting and, on the contrary, the least homogeneous the BG 27 sand.

### 3.2 Mechanical properties of tested mixtures

Transverse strength, abrasion resistance, loss on ignition and calculated relative porosity can be seen in Table 3 below. If transverse strength is considered as the main initial parameter, certain conclusions can be drawn. With increasing strength, abrasion resistance decreases direct proportionally, when the highest abrasion was measured for the 3DSP cores with a value of 16.31 % versus BG 21 with abrasion of 6.58 % and BG 27 with abrasion of 9.67 %. The finer the granulometric composition of the sand and the lower the d_50_ or the monofractional character of sand, the higher the strength was achieved, but this was valid only for the conventional method of compacting (BG 21 and BG 27). This statement was not valid in the case of cores produced by the 3Dsand print method due to higher relative porosity of the core, although the defined d_50_ of the sand was lower and sand is more monofractional. The lower core strength itself and high abrasion resistance could be the result of a given production technology.

**Table 3:** Measured parameters of tested sand cores.

Measurements performed	BG 21 sand core	BG 27 sand core	3D sand print
Transverse strength [MPa]	3.45	2.67	0.92
Bulk density of compacted core [g·cm ^−3^]	1.50	1.51	1.37
Abrasion [%]	6.58	9.09	16.31
Loss on Ignition [%]	1.14	1.12	1.20
Relative porosity [%]	43.18	42.80	48.11

3DSP cores showed the highest loss on ignition of 1.20 %, which is by 5–7 % higher than that one for BG 21 and BG 27 cores for which the same dosing of individual binder system components was used. This result corresponds to the expected and subsequently with the reverse method determined higher dosage of furan resin for the 3Dsand printed cores.

**Figure 9:**
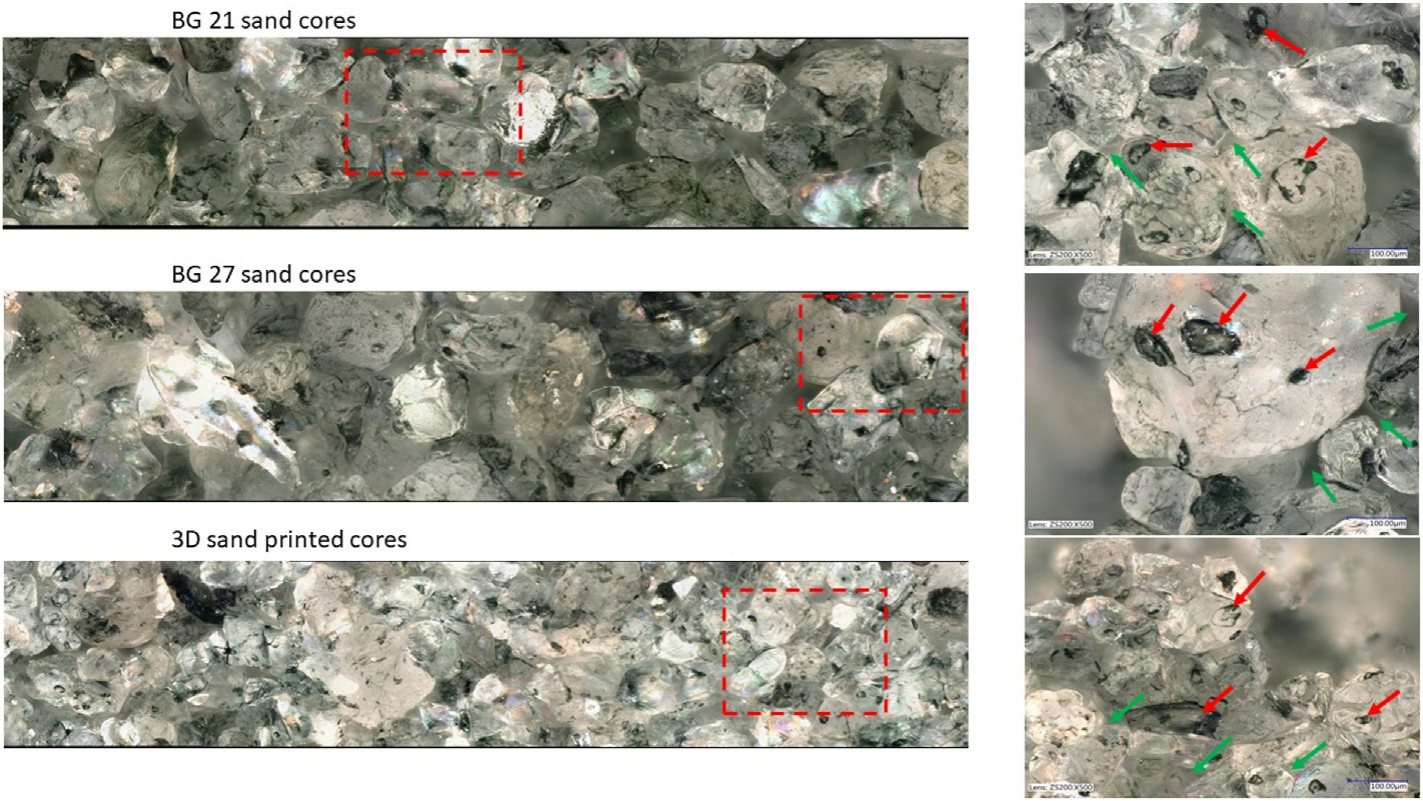
Detail of binder bridges (red arrows) and pores between grains (green arrows).

The relative porosity value of 48.11 % for the 3DSP cores compared with the BG 21 and BG 27 cores was by 11.4 % respectively 12.4 % higher. A detailed view of the inter-grain pores and binder bridges on the fracture surfaces of the cores after abrasion measurement is shown in Figure 9. The sand grains were purposely illuminated for greater visibility of the binder bridges. Photos were taken at 500x magnification on a digital microscope Keyence VHX 6000. Visually, the grains of all the samples can be assessed as angular shape. The contacts between the grains were therefore not point contacts, but were more like surface contact of different shapes. The porosity of the mixture does not depend on the diameter of the grains but on the structure of the mixture, i.e. the maximum possible number of contacts with neighbouring grains at a given degree of compaction, which was influenced not only by the shape of the grains but also by the composition of the mixture (monofraction and polyfraction). The distribution of pores between the grains corresponded with the calculated values of the relative porosity of the cores. In polyfraction mixtures, unlike monofraction, the addition of fines between the coarse fractions significantly reduces the absolute pore size of the mixture, which helps to prevent metal penetration into the mould. The finer fractions take the place of the coarser fractions in the intergranular spaces. The higher value for 3DSP cores is the result of fine monofractional sand used. Monofraction sands, on the other hand, have a lower total specific surface area and were therefore able to achieve higher strengths due to the formation of stronger bonding bridges when the binder was homogeneously distributed and compacted. Porosity and insufficient strengh can then be supported by unevenly applied individual layers of sand-catalyst mixture during the recoating process or too high recoater speed, where insufficient compression of the applied sand layer may occur. In both cases the result would be a low degree of compression of the mixture and thus a greater amount of pores between individual sand grains, which corresponds to the measured low bulk density of the compacted 3DSP core.

The results indicate there was no significant difference in loss on ignition, bulk density or relative porosity for BG 21 and BG 27 sand cores, and with regard to the same dosage of the binder and the catalyst and the production technology it was not even expected.

### 3.3 Determination of roughness of the core surface

**Figure 10:**
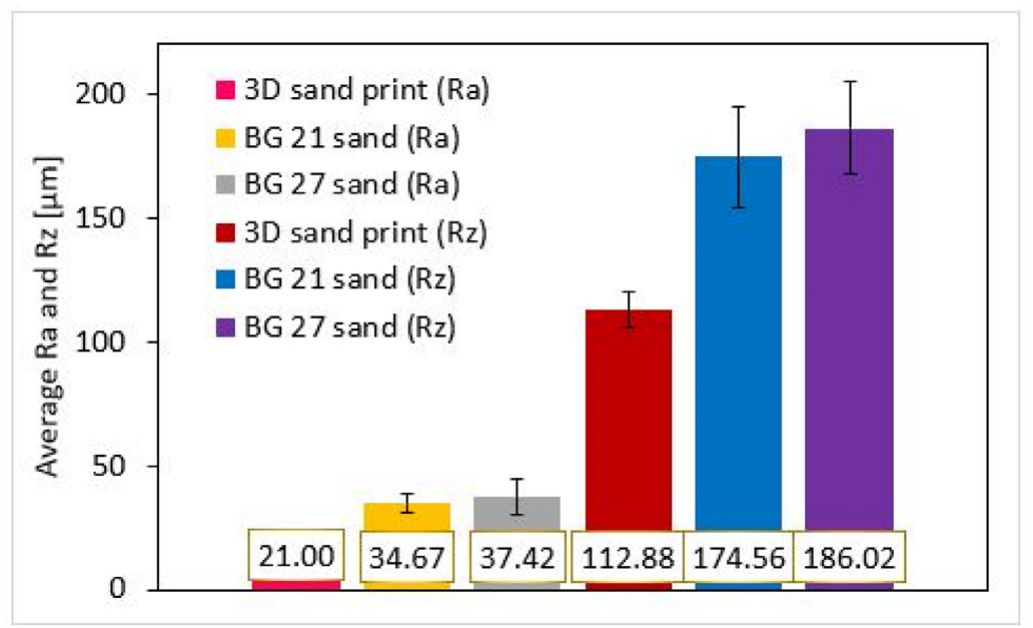
Roughness Ra and Rz of evaluated cores.

The roughness of the cores can be seen from Figure 10. 3DSP cores show the lowest surface roughness as expected as they were composed of sand grains with the finest d_50_ and a very sharply sorted nature of granulometric composition compared to furan no-bakes cores. The difference in surface roughness between the 3DSP and BG 21 cores was for Ra = 13.67 μm and Rz = 61.68 μm. The difference in roughness between 3DSP and BG 27 cores was for Ra = 16.42 μm and Rz = 73.14 μm. The largest measured differences in roughness correspond to differences between the highest and lowest d_50_ for the sands used, i.e. for 3DSP and BG 27 samples. In contrast, BG 21 and BG 27 cores do not show such significant differences in roughness as might be expected given the different sizes of the mean grain size d_50_. The sands used for these cores did not differ that much from each other in the distribution of grain fractions on the sieves. The total measured differences in roughness between BG 21 and BG 27 cores were for Ra = 2.75 μm and Rz = 11.46 μm. Consistent surface roughnesses were measured on all core surfaces.

### 3.4 Determination of the casting roughness

During the filling of the mould cavity with liquid metal, the flow causes all the impurities and inclusions to float to the top surface of the casting or to be trapped on the bottom surface of the core. This leads to a higher surface quality on the lower surfaces than on the upper surfaces with deposited impurities. The same is also applicable in the case of the inner cavities of castings formed by cores with the upper inner surface (see Figure 6) impurities and an increased surface roughness by loose grains from the core mixture can be found.

**Figure 11:**
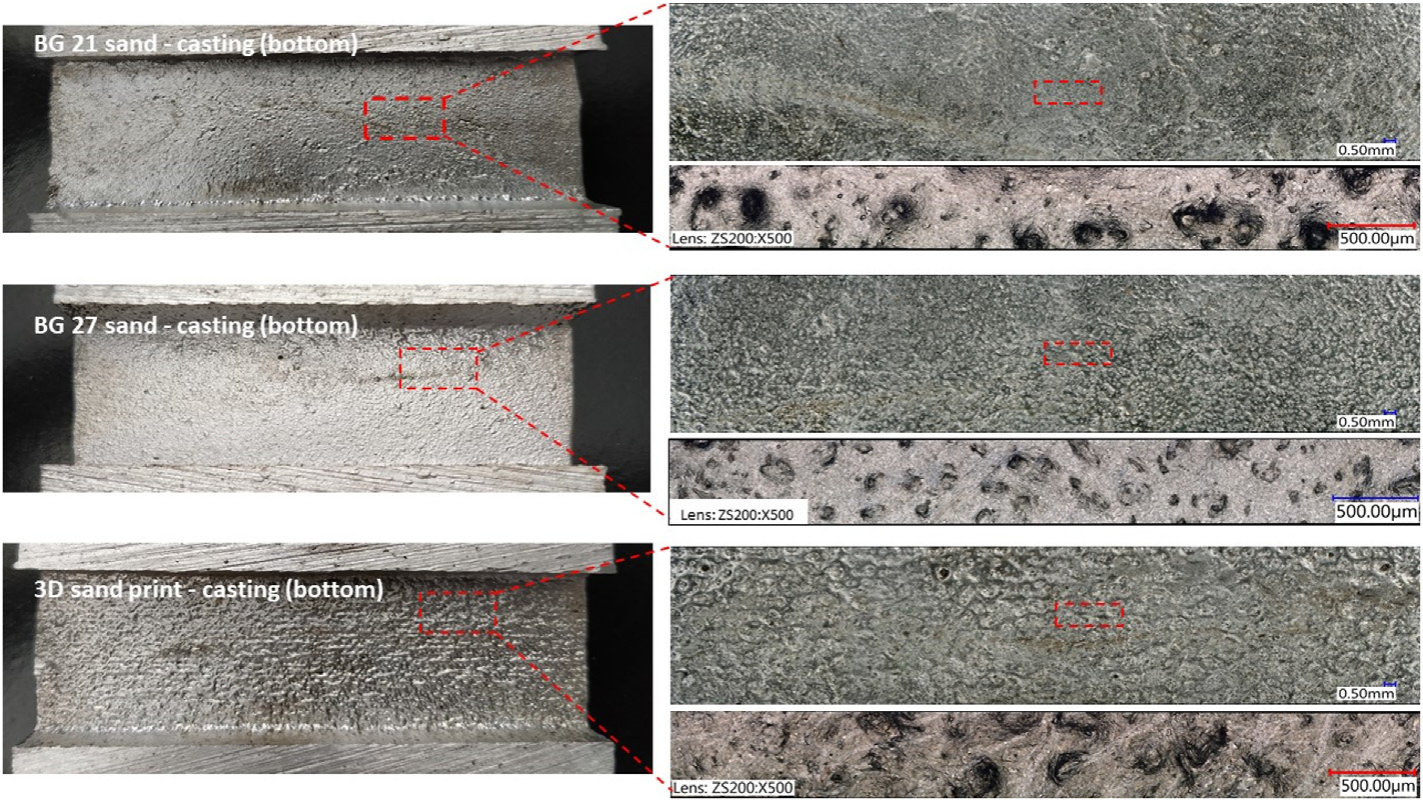
Surface roughness at casted condition made by cores from sand BG 21, BG 27 and 3D sand printed.

**Figure 12:**
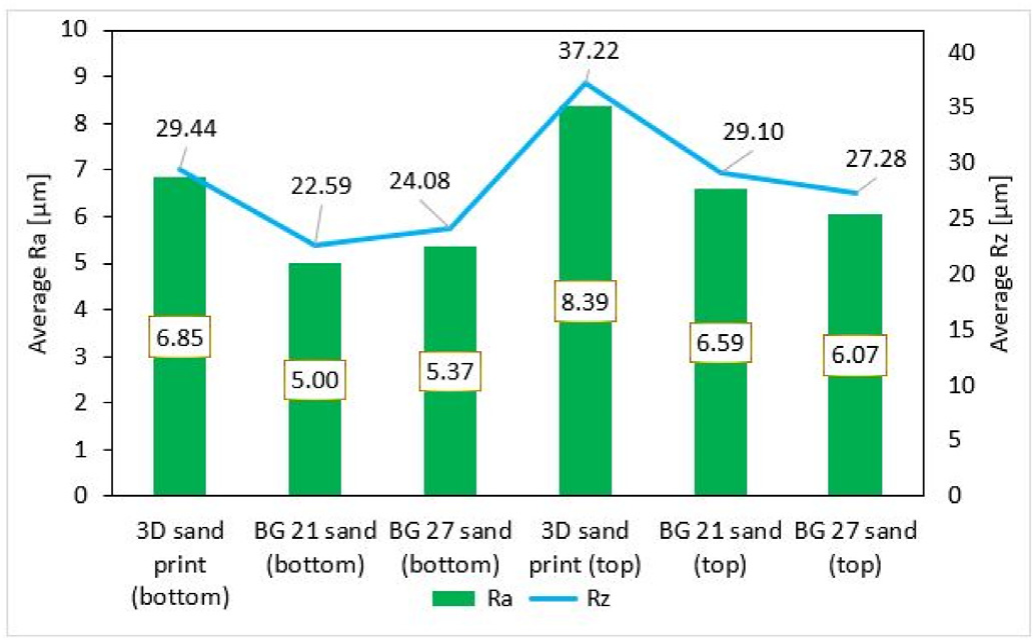
Roughness Ra and Rz of the surfaces of the top and bottom inner part of the casting.

The roughness of the castings are shown in Figure 11, where the designation 3DSP was for cores made by 3D sand printing and BG 21, BG 27 for furan no-bake cores. Pictures show the roughness of area marked as “bottom”, made by upper part of the core. In the photos with the highest magnification (500x), large cavities were visible. These were not surface porosity or gas defects of the casting, but the imprint of the individual grains of the sand at high depth image. At first glance, the difference between casting areas copying 3DSP cores was noticeable. Figure 12 shows, that in the case of the “bottom” inner side of the casting, the roughness difference between the 3DSP core and the BG 21 core was Ra = 1.85 μm, Rz = 6.85 μm. There was a difference of Ra = 1.48 μm and Rz = 5.36 μm between the 3DSP and the BG 27 one. For the furan no-bake (the BG 21 and the BG 27), the difference was Ra = 0.37 μm and Rz = 1,49 μm.

The “top” inner surface of the casting area formed from the core side shows the same trend, namely increased surface roughness in the case of 3DSP cores. The differences were as follows: 3DSP and BG 21 the difference was Ra = 1.8 μm and Rz = 8.12 μm, 3DSP and BG 27 Ra = 2.32 μm and Rz = 9.94 μm. The differentiation between BG 21 and BG 27 was Ra = 0.52 μm and Rz = 1.82 μm only and it can be concluded that the grain size in this case does not have a significant influence on surface quality.

**Figure 13:**
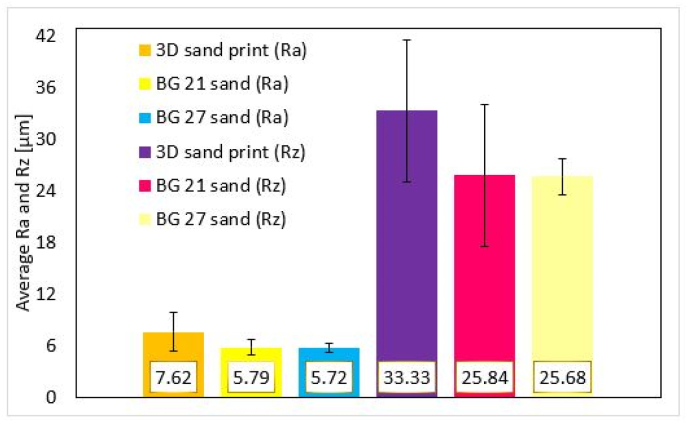
Overall surface roughness Ra and Rz of castings.

By averaging the values of roughness of the top and bottom inner part of the casting, an overall view on the achieved surface quality of the product was obtained (see Figure 13). Obtained results of the casting surfaces (average Top and Bottom values) were lower when using cores produced by the 3D printing method. In the case of areas marked 3DSP vs BG 21 the value was decreased by Ra = 1.83 μm and Rz = 7.49 μm. The differences between the area of 3DSP and BG 27 were almost identical (the decrease Ra = 1.9 μm and the decrease Rz = 7.65 μm). The resulting values were approximately the same for areas where BG 21 and BG 27 cores were used, respectively negligible differences Ra = 0.007 μm and Rz = 0.16 μm. Thus, the achieved results indicate a slight deterioration of the surface quality of the areas where the cores produced by the 3D printing method were used. If evaluated the average surface quality between BG 21 and BG 27 areas, it can be considered that the size of the grains of the used sands does not cause a significant decrease of surface quality in total.

## 4 Discussion

Both, the roughness of the core surfaces and the resulting roughness of the surfaces of casting cavities formed from cores were compared in this study. The results of this measurement correspond to the measured mechanical properties of furan no-bake cores and the 3DSP technology.

Cores produced by the 3DSP technology compared to the other tested sands, BG 21 and BG 27, were made using very homogenous sand of high fineness, with sharply sorted fractions and an overall granulometric composition approaching rather to monofraction, as confirmed by the lowest measured log W value of 41.49 and the highest homogeneity degree S of 68.75 %. As expected, the 3D sand printed cores itself show significantly lower surface roughness. In contrast, the resulting surface of the casting cavities showed the highest roughness and overall poorer quality of the cast surface. The total average measured difference for casting cavity surfaces formed from cores was for 3D sand print Ra by 31.6 % higher compared to the BG 21 and by 33.2 % respectively compared to BG 27. Rz roughness was by 28.99 % higher in case of 3Dsand print compared to the BG 21 and by 29.79 % compared to the BG 27. The differences in roughness between the BG 21 and the BG 27 cores were Ra 1.22 % and Rz 0.62 % for casting cavity surfaces and therefore those were almost negligible. None of the surfaces of the made castings showed surface defects or cavities of a gas nature.

Contrary to expectations, the results obtained for the roughness of the internal surfaces of samples BG 21 and BG 27 were different. However, the measured results show a rougher internal surface of the casting in the case of BG 21 cores, although the overall difference in roughness Ra and Rz was only minimal. From the results, it can be concluded that the overall homogeneity and the distribution of the individual sand fractions on the sieves of both samples have a great influence on the resulting surface quality of the casting. Despite the fact that the BG 27 cores were composed of sand with a coarser d_50_ (0.23 mm) than BG 21 (0.19 mm), the sand in the case of BG 27 was more heterogeneous in composition with a cumulative curve of granularity showing a more polyfractional character (Figure 8) than was the case for BG 21 sand, which was more homogeneous with a greater volume of grains in several individual fractions. Also, the calculated relative porosity of the core is higher in the case of BG 21 by 0.89 % compared to BG 27. Thus, it can be seen that the sands having a rather heterogeneous and polyfractional character, and thus containing both coarse and very fine particles in similar amounts, contribute to the resulting smoothness of the cast surface. This was caused by the ability of the fine particles to fill the intergranular spaces between the coarse sand grains, making it difficult for liquid metal to penetrate and copy the grain shape and pores. This could be seen from Figure 11 (the highest magnification), where the smoothest surface with fewer deep sand grain impressions was observed in BG 27 samples.

Worse casting surface quality in case of 3DSP cores was also indicated by the measured mechanical properties of the cores, where the 3DSP cores showed extremely low strength (transverse strength) compared to the strength declared by the manufacturer (∼4 MPa) and namely only 0.92 MPa on average compared to 3.45 MPa for the BG 21 and to 2.67 MPa for the BG 27. Then the results of other tested properties correspond with these strengths, when a very low bulk density was measured for the 3DSP cores, as well as high abrasion, relative porosity and loss on ignition resulting in disturbing of surface layer of the core during pouring of liquid metal a deterioration of quality of the cast surface.

The approximate used furan content in the 3DSP cores was determined retrospectively from the loss on ignition. The observed furan dosage was higher than the conventional furan no-bake method of core production, but not as high as commonly reported. The effect of the dosage of furan binder and the setting of optimum printing parameters was also shown in a study by another author [36], where when the binder content was reduced during 3DSP, the pieces recorded the best surface quality in terms of pitting and gas porosity, but the cores produced in this way achieved low strengths. Possible explanations for the insufficient mechanical properties of the cores and the high roughness and low quality of the cast surface can be several, or a combination thereof as follows:

• Compared to the dosing of furan generally given in the literature, the determined dosage of furan in the case of these specific 3DSP cores could be in real process lower than required. This may be the reason for the lower binder capabilities of the mixture and thus for the lower core strengths and related properties. In principle, the binder must be able to harden between the individual layers of the applied mixture in such a way as to prevent the new layer from spreading out on still moist particles. This usually leads to the use of excess binder for 3D sand printing.
• The use of lower quality furan resin with lower furfuryl alcohol content
• The use of very fine and sharply sorted sand of very low heterogeneity. This can lead to a higher number of pores in the mixture and higher requirements for sufficient dosage of binder, the lack of which can lead to reduced strength and this a deteriorated casting surface.
• Too high print speed and therefore recoater speed too, causing uneven application of a new layer of sand with a catalyst and insufficient compression of the applied layer. The result was a low compaction of the mixture and the formation of free intergrain spaces and pores in the applied layer of sand, which can be further supported by the choice of sharply sorted sand with little or no representation of fine fractions of the sand.
• Too high print speed and therefore uneven dosing of furan in the form of small droplets, which should be evenly applied by the print head. Considering the possible larger area of pores between the individual grains of the moulding mixture, as well as between the individual layers, there was also the risk of entering of a part of the binder into the lower layer and the formation of an imperfectly hardened upper layer.

## 5 Conclusions

The cores tested were produced using furan no-bake technology and 3D sand printing. The granulometric composition of the basic sand was evaluated as well as the mechanical properties of the cores. The obtained cores were used for the production of castings to determine the resulting surface roughness in the cast state. The main conclusions are as follows:

• Cores produced by3D sand print have low surface roughness as a result of a finer mean grain size d_50_ compared to conventional furan no-bake technology.
• 3D sand printed cores achieve significantly lower strength (0.92 MPa) than furan no-bake cores (3.45 MPa), 2.5 times higher abrasion, 5–7 % higher loss on ignition and 12.4 % higher relative porosity than conventional furan no-bake cores.
• The resulting Ra roughness of the cast surface in the case of 3D printed cores was 33.2 % higher than in the case of furan no-bake technology as a result of low mechanical properties of printed cores.
• No significant differences in surface roughness were observed when using cores of silica sands BG 21 and BG 27 with different mean grain size d_50_.
• No gas defects or porosity were observed on the surface of the castings.

## Declarations

### Author contribution statement

Martina Gawronová: Performed the experiments; Analyzed and interpreted the data; Wrote the paper.

Petr Lichý, Jan Jeziersk: Conceived and designed the experiments.

Ivana Kroupová: Performed the experiments; Wrote the paper.

Tomáš Obzina, Václav Merta: Performed the experiments.

Jaroslav Beňo, Filip Radkovský: Analyzed and interpreted the data.

Isabel Nguyenová: Contributed reagents, materials, analysis tools or data.

### Funding statement

This work was supported by 10.13039/501100001823Ministerstvo Školství, Mládeže a Tělovýchovy (CZ.02.1.01/0.0/0.0/17_049/0008399). This work was carried out in the support of projects by Ministerstvo Školství, Mládeže a Tělovýchovy ​ “Student Grant Competition” SP2022/15 and SP2022/83.

### Data availability statement

The authors do not have permission to share data.

### Declaration of interests statement

The authors declare no conflict of interest.

### Additional information

No additional information is available for this paper.
